# Surface-Engineered Manganese Oxide via Sodium Borohydride for Optimized ORR Active Electrocatalyst

**DOI:** 10.1021/acsomega.5c08148

**Published:** 2025-10-23

**Authors:** Jithul KP, Jay Pandey

**Affiliations:** Department of Chemical Engineering, Birla Institute of Technology & Science Pilani, Pilani Jhunjhunu 333031, Rajasthan, India

## Abstract

Manganese oxide octahedral molecular sieves (OMS) have garnered attention as promising electrocatalysts for oxygen reduction reactions (ORRs) due to their cost-effectiveness as well as durability. However, their practical application is limited by inherent drawbacks such as low electrical conductivity and insufficient intrinsic catalytic activity. To overcome these challenges, we employed a surface reduction etching treatment using NaBH_4_ to optimize the oxygen vacancy of OMS. The treatment with a 6 mmol/L NaBH_4_ solution significantly increased the number of oxygen vacancies on the surface of OMS, which serve as crucial active sites facilitating the adsorption and dissociation of oxygen molecules, thereby enhancing ORR activity. Furthermore, the treatment effectively regulated the Mn^3+^/Mn^4+^ ratio on the nanosphere surface, further promoting catalytic efficiency by facilitating the transfer of electrons during the ORR process. Notably, the optimized OMS material exhibited a remarkable half-wave potential of 0.661 V, highlighting its improved performance and potential as a suitable replacement for traditional platinum-based catalysts. This straightforward and scalable method unlocks the potential of OMS materials for practical applications, offering a promising solution for energy storage as well as conversion technologies that require efficient ORR catalysts.

## Graphical abstract

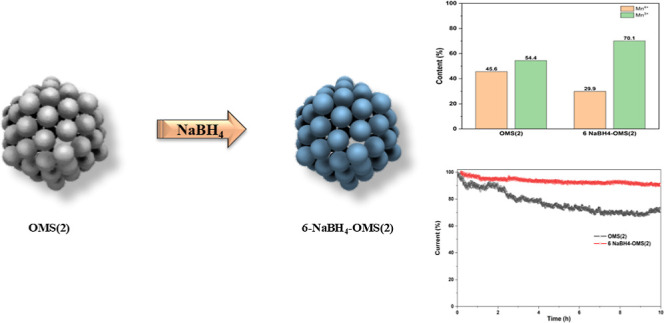

## 1 Introduction

The current energy crisis has spurred intensive research into sustainable alternatives to conventional energy sources, with fuel cells emerging as a particularly promising technology. However, widespread commercialization of fuel cells remains hampered by significant cost barriers. The efficiency of the cathode is hindered by the slow reaction of the oxygen reduction reaction (ORR), a complex, multielectron process that critically impacts fuel cell performance and durability.^1–4^ The reliance on expensive, platinum-based catalysts for the ORR further compounds the cost challenge. Consequently, substantial research efforts are focused on developing high-performance, nonprecious metal ORR catalysts. Among the various candidates, manganese oxides have attracted considerable interest due to their inherent advantages, including multiple oxidation states, structural diversity, natural abundance, and low toxicity. Crucially, the catalyst activity of manganese oxides (MnO_*x*_) for the ORR is strongly dependent on their electronic structure, which is intimately related to both the manganese oxidation state and crystalline phase. Therefore, controlled synthesis and characterization of manganese oxides with tailored valence states and crystal structures are essential for optimizing their ORR performance and fully leveraging the advantages of fuel cell technology.^5–8^

Manganese oxide catalytic activity for ORR is fundamentally limited by two key factors: oxygen adsorption/desorption kinetics and electrical conductivity. The ORR involves a complex interplay of oxygen and oxygen-containing species, requiring optimized oxygen binding energy at the manganese active site to facilitate efficient and reversible oxygen adsorption.^9^ Furthermore, the typically low intrinsic conductivity of manganese oxides hinders efficient electron transfer during the multielectron ORR process. While various strategies, including heteroatom doping, compositing with conductive materials, and advanced surface modification techniques like etching, have been explored to address these limitations, the controlled introduction of oxygen vacancies has emerged as a particularly promising and versatile approach.^10,11^ Oxygen vacancies, as point defects in the lattice structure, significantly perturb the electronic structure and distribution within the material, thereby directly influencing its catalytic properties.^12–14^ These defects can enhance the intrinsic conductivity by providing additional charge carriers, facilitating electron transport and promoting ORR kinetics. Critically, surface oxygen vacancies can also modify the chemical environment of the catalytically active sites, influencing the intermediate’s adsorption energies of oxygen and optimizing the overall catalytic process.^15^ Therefore, the strategic engineering of oxygen vacancy concentrations and distributions represents a compelling pathway for enhancing the ORR performance of manganese oxide-based electrocatalysts. In summary, this study explores the controlled generation of oxygen vacancies in manganese oxide octahedral molecular sieves (OMS(2)) through a novel approach combining sodium borohydride (NaBH_4_) etching and vacuum annealing. By precisely modulating the NaBH_4_ concentration, we aim to achieve fine control over the oxygen vacancy content, thereby optimizing the electrocatalytic activity as well as stability of the resulting materials for the ORR. A detailed exploration of the catalytic mechanism and the correlation between oxygen vacancy concentration and performance will provide valuable insights for the design of efficient electrocatalysts.^16–21^

## 2 Experimental Section

### 2.1 Materials and Chemicals

Manganese­(II) acetate tetrahydrate (Mn­(CH_3_COO)_2_·4H_2_O), potassium permanganate (KMnO_4_), sodium borohydride (NaBH_4_), commercial Pt/C (10 wt %, sigma aldrich), and 5 wt % Nafion solution.

### 2.2 Synthesis of OMS(2)

A hydrothermal process was employed to create OMS(2). A solution was prepared by dissolving Manganese­(II) acetate tetrahydrate (Mn­(CH_3_COO)_2_·4H_2_O) and potassium permanganate in a 1:2 molar ratio. To achieve a uniform mixture, stirring was conducted for 30 min. Subsequently, the solution was kept in a sealed autoclave and heated at 140 °C for 24 h under hydrothermal conditions. Following the reaction, the resulting solid product was recovered and subjected to repeated washing steps to remove any unreacted precursors or byproducts. The washed material was then dried overnight at 80 °C.

### 2.3 Synthesis of NaBH_4_–OMS­(2)

A series of sodium borohydride-treated OMS-2 catalysts were prepared by reacting 1 g of the synthesized OMS(2) with 50 mL of NaBH_4_ solutions at varying concentrations (3, 6, 9, and 12 mmol/L). The reactions were conducted under continuous stirring for 30 min. Following the reaction period, the resulting materials were immediately separated via centrifugation. The washed materials were subsequently dried at 60 °C. Finally, the dried products underwent vacuum annealing at 200 °C for 2 h (Scheme 1). These treated materials are designated as X NaBH_4_–OMS­(2) catalysts, where X represents the concentration of the NaBH_4_ solution used in the treatment.

**Scheme 1:**
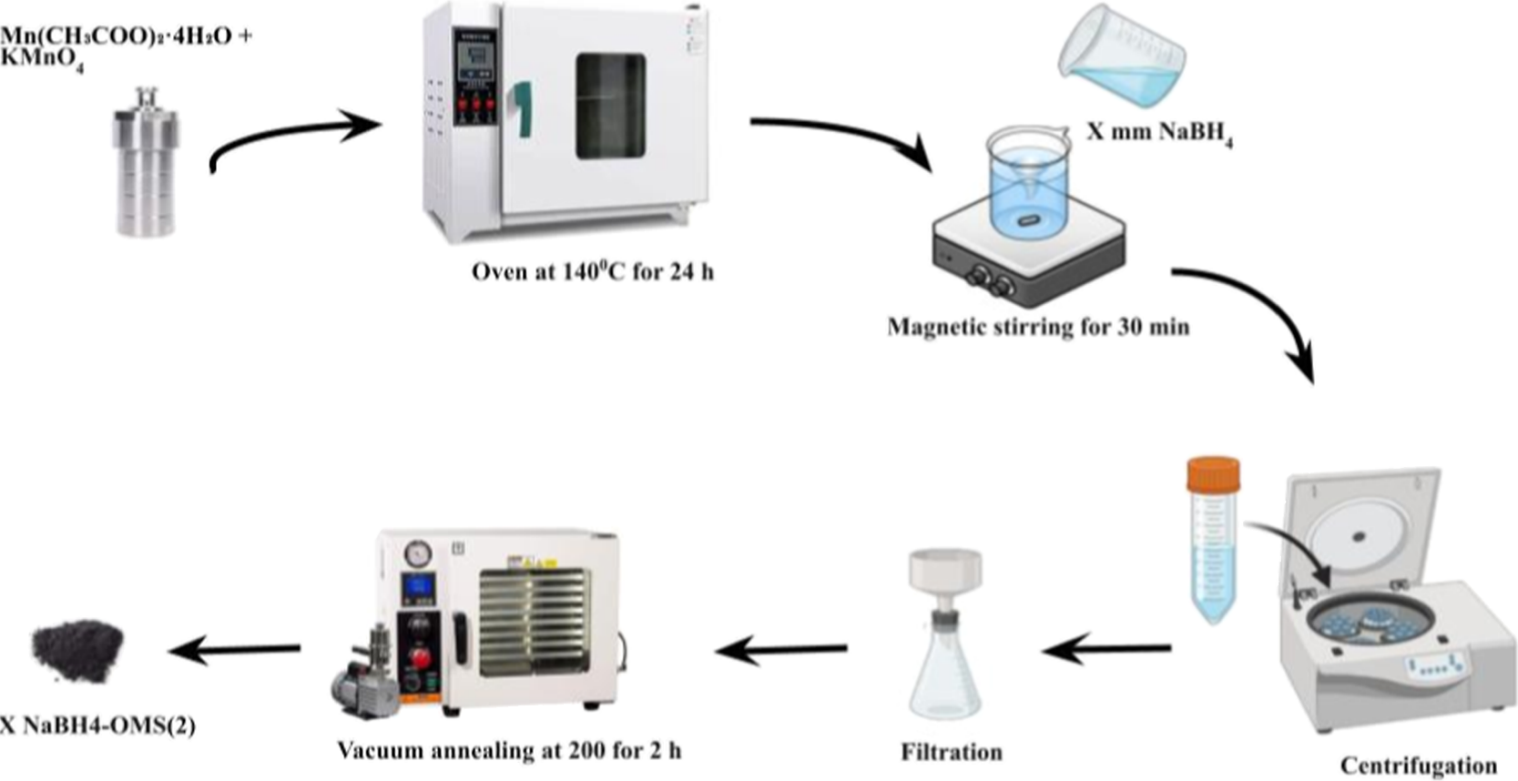
Schematic Illustration of NaBH_4_–OMS­(2) Electrocatalyst Synthesis

### 2.4 Preparation of Sample Electrodes

To prepare the electrodes, 5 mg of synthesized catalysts were dispersed in a mixture of 250 μL of ethanol, 700 μL of Milli-Q water, and 50 μL of Nafion. After ultrasonication for 30 min, 10 μL of ink was drop cast over 1 cm × 1 cm Ni foam, loading 0.6 mg cm^–2^. In the same way, a 10 wt % Pt/C electrode is also drop cast over a 1 cm × 1 cm Ni foam. One set of 6 NaBH_4_–OMS­(2) was drop cast over glassy carbon electrodes (3 mm diameter, surface area = 0.71 cm^2^, loading 0.5 mg cm^–2^) for RDE.

## 3 Results and Discussion

As shown in Scheme 1, the synthesis of the NaBH_4_-treated OMS­(2) electrocatalyst involves a treatment with NaBH_4_, which reduces Mn^4+^ to Mn^3+^ and creates oxygen vacancies within the lattice while oxidizing BH_4_^–^ ions to borate byproducts. However, this chemical reduction alone is insufficient, as it leaves behind surface-adsorbed water, residual borates, and unstable defect sites. Therefore, a subsequent vacuum annealing step (200 °C for 2 h) is indispensable. This thermal treatment is critical for achieving a high-performance catalyst, serving the dual purposes of purification (removing adsorbates) and structural stabilization. XRD analysis was performed to investigate the crystal structure of the synthesized OMS(2) and the impact of the sodium borohydride (NaBH_4_) treatment. Figure 1a illustrates the XRD patterns of the synthesized OMS(2) and the NaBH_4_-treated OMS(2) samples at varying NaBH_4_ concentrations (3, 6, 9, and 12 mmol/L). The diffraction pattern of the pristine OMS(2) exhibited characteristic peaks consistent with the 2 × 2 tunnel structure of α-MnO_2_, matching the reference pattern (JCPDS No. 44-0141).^22^ This confirmed the successful formation of OMS(2) via the hydrothermal synthesis method. Following the NaBH_4_ treatment, all samples (denoted as *X* NaBH_4_–OMS­(2)) retained the characteristic peaks of the OMS(2) structure, indicating the structural integrity of the OMS(2) framework. Specifically, peaks observed at, 18.10°, 29°, 32.7°, 37.52°, 45°, 50.9°, 54°, 58.6° and 60° correspond to the (200), (310), (400), (211), (510), (411), (440), (600) and (521) planes of α-MnO_2_, respectively, and were present across all samples.^23^ Upon increasing the NaBH_4_ concentration, a trend of decreasing peak intensity was observed. However, a more pronounced decrease in peak intensity was observed for the sample treated with the highest NaBH_4_ concentrations (9, 12 mmol/L). This suggests that the higher NaBH_4_ concentration induces more significant structural changes, potentially due to an etching effect that selectively removes surface atoms. This leads to a reduction in the overall surface crystallinity of the material and the creation of defects, consistent with the observed broadening and lower intensity of the diffraction peaks.

**Figure 1:**
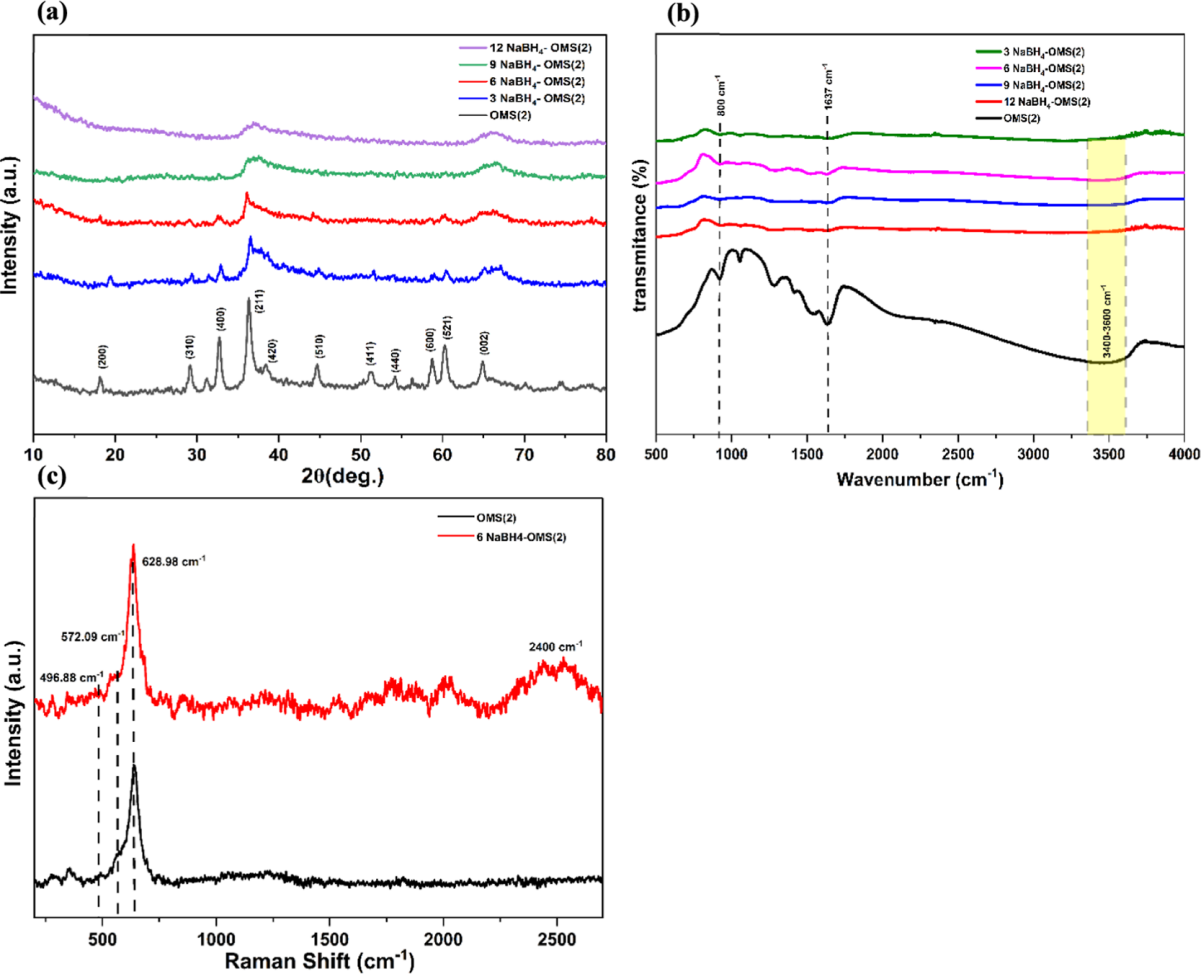
(a) XRD, (b) FTIR of all electrocatalysts, (c) Raman of OMS (2) and 6 NaBH_4_–OMS­(2) electrocatalysts.

Figure 1b presents the FTIR spectra of the pristine OMS(2) and the NaBH_4_-treated electrocatalyst. Spectral analysis revealed characteristic vibrational modes indicative of the material’s composition and surface functionalities. A distinct vibrational mode was observed at approximately 800 cm^–1^, corresponding to the lattice vibration of Mn–O within the α-MnO_2_ octahedral framework. This confirms the presence of the desired manganese dioxide phase in both samples. Prominent peaks at approximately 1637 cm^–1^ and 3400–3600 cm^–1^ were identified, which are attributed to the bending and stretching vibrations of adsorbed water molecules on the electrocatalyst surface, respectively. These peaks are commonly observed in metal oxide materials due to their hydrophilic nature. A notable reduction in the intensity of all three characteristic peaks (800 cm^–1^, 1637 cm^–1^, and 3400–3600 cm^–1^) was observed in the NaBH_4_-treated electrocatalyst compared to the pristine OMS(2).^24^ This suggests a decrease in both the Mn–O lattice vibration intensity and the surface-adsorbed water content. Furthermore, a slight shift of the Mn–O vibration peak toward lower wavenumbers (left shift) was observed in the NaBH_4_-treated sample. This shift indicates a weakening of the Mn–O lattice vibrational force, potentially due to structural modifications induced by the NaBH_4_ treatment. The observed weakening and slight shift of the Mn–O vibration peak toward lower wavenumbers in the NaBH_4_-treated sample (from 800 cm^–1^) suggest a weakening of the Mn–O lattice bonds. This weakening is consistent with the removal of lattice oxygen atoms and the creation of structural defects, i.e., oxygen vacancies. The reduced intensity of the broad peak at the range of 3400–3600 cm^–1^ and 1637 cm^–1^, corresponding to adsorbed water, is likely due to the additional vacuum annealing step that was performed after the NaBH_4_ treatment. This additional step would remove much of the adsorbed water. The observed weakening of the Mn–O lattice vibration suggests that the NaBH_4_ treatment may induce structural changes in the α-MnO_2_ framework, potentially involving the introduction of oxygen vacancies or the alteration of Mn-oxidation states. The Raman spectra of both OMS(2) and 6 NaBH_4_–OMS­(2) displayed characteristic peaks corresponding to the Mn–O vibrational modes within the MnO_2_ octahedral units­(Figure 1c). Specifically, these peaks are observed at Raman shifts of 496.88 cm^–1^, 527.09 cm^–1^, and 628.98 cm^–1^, which align with the spectral signature of pure OMS(2). The NaBH_4_–OMS­(2) exhibited stretching vibration of the B–H bond in BH_4_^–^ ions at 2400 cm^–1^, which confirms the successful incorporation of NaBH_4_ in OMS(2).^25^

The surface morphology and microstructure of the pristine OMS(2) and the optimized 6 NaBH_4_–OMS­(2) electrocatalysts were investigated using Field Emission Scanning Electron Microscopy (FESEM), as shown in Figure 2. The pristine OMS(2) material (Figure 2a) consists of relatively uniform nanospheres with a calculated average diameter of 69.5 nm (Figure S1). Following the chemical reduction with NaBH_4_, the overall nanosphere morphology was well-preserved in the 6 NaBH_4_–OMS­(2) sample (Figure 2c). However, a discernible increase in the average particle diameter to 80.3 nm was observed (Figure S2), accompanied by the formation of a rougher, more exposed surface texture. This morphological evolution is attributed to the surface etching effect of the NaBH_4_ treatment, which modifies the nanostructure.^23,26^ To complement the morphological analysis, EDS was employed to map the elemental composition, as presented in Figure 2b,d. Both the pristine and treated samples show a uniform distribution of Mn and O throughout the nanosphere framework. Critically, a quantitative analysis of the EDS data revealed a significant decrease in the oxygen atomic percentage from 22.37% in the pristine OMS(2) to 14.40% in the 6 NaBH_4_–OMS­(2) (Table S1). This substantial reduction in oxygen content provides direct and compelling evidence for the successful creation of oxygen vacancies within the catalyst structure, which is the primary goal of the NaBH_4_ reduction.^23^ HRTEM was utilized to probe the atomic-scale structure and crystallinity of the optimized 6 NaBH_4_–OMS­(2) catalyst. The TEM image in Figure 2e further corroborates the nanospherical morphology observed in FESEM. The HRTEM images (Figure 2f,g) reveal well-defined lattice fringes, indicating the high crystallinity of the material. The measured interplanar spacing was 0.312 nm, which corresponds precisely to the (310) crystal plane of the cryptomelane-type manganese oxide octahedral molecular sieve structure. This result confirms that the crystalline framework of the catalyst remains intact and well-ordered, even after undergoing the chemical reduction process designed to introduce a high density of beneficial oxygen vacancies.^27^

**Figure 2:**
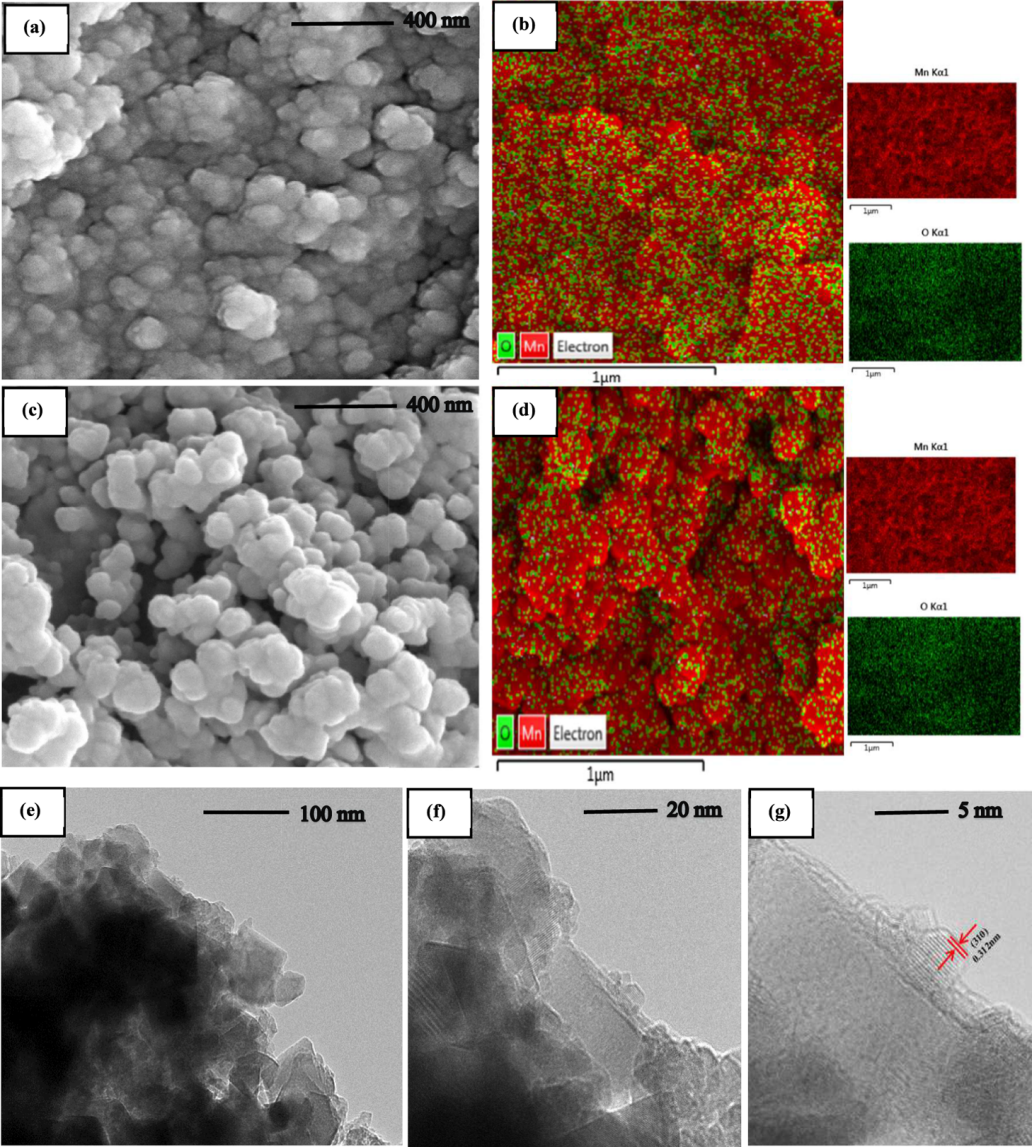
FESEM image of (a) OMS(2) (c) 6 NaBH_4_–OMS­(2) electrocatalysts. EDS elemental mapping of (b) OMS(2) (d) 6 NaBH_4_–OMS­(2) electrocatalysts. Six NaBH_4_–OMS­(2) electrocatalyst (e) TEM (f,g) HRTEM.

XPS analysis of the O 1s core level revealed three distinct spectral features at binding energies of 533.6, 532.2, and 530.8 eV, corresponding to chemisorbed oxygen (O_3_), oxygen vacancies (O_2_), and lattice oxygen (O_1_), respectively. Notably, the NaBH_4_-treated OMS(2) sample exhibited a significant enhancement in the O_2_ peak intensity, indicative of an increased concentration of oxygen vacancies compared to the pristine OMS(2) material (Figure 3a). Furthermore, the O 1s peak for lattice oxygen (O_1_) shifts negatively (to lower binding energy) for the NaBH_4_-treated sample compared to the pristine OMS(2). This negative shift is a consequence of the reduced average positive charge of neighboring manganese ions (Mn^4+^ is reduced to Mn^3+^) upon vacancy formation, which increases the electron density around the remaining lattice oxygen atoms, thereby enhancing nuclear shielding. The Mn 2p core level spectra (Figure 3b) displayed spin–orbit splitting, with a separation of 11.5 eV between the Mn 2p^3/2^ and Mn 2p^1/2^ peaks, confirming the coexistence of Mn^3+^ and Mn^4+^ oxidation states. Deconvolution of the Mn 2p spectra enabled the precise determination of the binding energies associated with Mn^3+^ (641.2 and 653.2 eV) and Mn^4+^ (642.4 and 654.1 eV) species.^28–30^ Interestingly, the Mn^4+^ peak of the NaBH_4_-treated sample undergoes a positive shift (to higher binding energy), an effect opposite to the O 1s shift. This shift is attributed to the removal of a highly electronegative neighboring oxygen atom (O^2–^), which decreases the electron density surrounding the remaining Mn^4+^ ions, thus reducing nuclear shielding and requiring more energy for core electron ejection. Quantitative analysis (Figure 3c) demonstrated a direct correlation between the NaBH_4_ treatment concentration and the relative abundance of Mn^3+^, with the Mn^3+^/Mn^4+^ ratio increasing progressively. Specifically, the relative Mn^3+^ content increased from 54.4% in the pristine sample to 70.1% in the treated sample, accompanied by a corresponding decrease in Mn^4+^ content from 45.6% to 29.9%. This observed increase in Mn^3+^ concentration is consistent with the observed augmentation in oxygen vacancy density, a phenomenon attributable to the need to maintain charge neutrality within the material.^31^ The formation of oxygen vacancies necessitates the reduction of Mn^4+^ to Mn^3+^ to compensate for the loss of negatively charged oxygen ions. The concordant trends observed for oxygen vacancy concentration and Mn^3+^ content underscore the efficacy of NaBH_4_ treatment in modulating the electronic as well as structural properties of OMS(2). These modifications, particularly the creation of oxygen vacancies and the alteration of the Mn^3+^/Mn^4+^ redox couple, are anticipated to play a pivotal role in the ORR performance enhancement, as oxygen vacancies serve as active sites for the adsorption of O_2_ and activation, and the Mn^3+^/Mn^4+^ redox transition facilitates electron transfer during the ORR.

**Figure 3:**
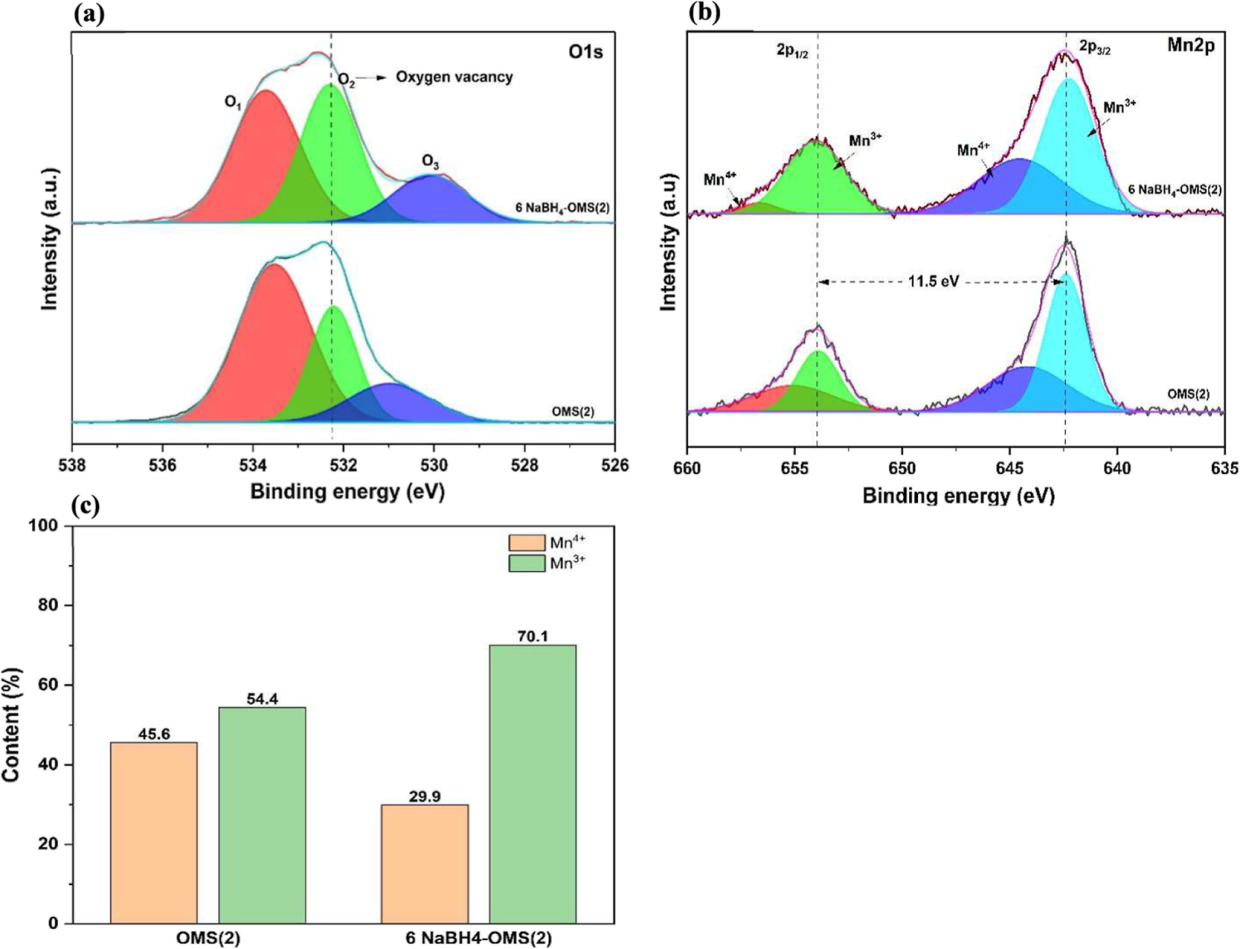
XPS image of (a) O 1s, (b) Mn 2p (c) relative content of Mn^3+^ and Mn^4+^ of OMS(2) and 6 NaBH_4_–OMS­(2) electrocatalysts.

Electrochemical evaluation of OMS(2) catalysts, subjected to varying concentrations of sodium borohydride (NaBH_4_) to modulate oxygen vacancy defects, revealed a significant impact on ORR performance. Linear sweep voltammetry (LSV) conducted at a scan rate of 5 mVs^–1^ (as shown in Figure 4a), demonstrated that 6 NaBH_4_–OMS­(2) exhibited the highest current density (6 mA cm^–2^), indicating superior ORR activity. This catalyst showed a significantly improved half-wave potential (*E*_1_/_2_) of 0.661 V vs RHE, a 78 mV positive shift compared to pristine OMS(2). At this potential, the current density is 2.44 mA cm^–2^ and the onset potential (*E*_onset_) is 1.06 V vs RHE. The *E*_1/2_ and *E*_onset_ values are comparable with state-of-the-art 10 wt % Pt/C results (Table S2). This highlights the beneficial role of optimized oxygen vacancy concentration in enhancing ORR kinetics. Both lower (3 NaBH_4_–OMS­(2)) and higher (9 and 12 NaBH_4_–OMS­(2)) NaBH_4_ treatments resulted in decreased performance, suggesting that both insufficient and excessive oxygen vacancy creation negatively impact catalyst effectiveness. The absence of a proper limiting current plateau is characteristic of measurements on stationary, porous electrodes like Ni foam, where mass transport is dominated by natural convection and complex diffusion pathways often lead to mixed kinetic-diffusion control.^32^ Tafel plot analysis in Figure 4b further corroborated these findings, revealing that 6 NaBH_4_–OMS­(2) displayed the lowest Tafel slope (38.8 mV dec^–1^) comparable with Pt/C Tafel slope (29.1 mV dec^–1^), signifying the most favorable reaction kinetics, in contrast to the steeper slope of pristine OMS(2), indicative of slower kinetics.^33,34^ CVs for both O_2_- and N_2_-saturated solutions (as shown in Figure 4c) provide a clear understanding of ORR behavior. N_2_-saturated solutions served as a baseline with no significant cathodic current, which confirm the absence of ORR while O_2_- saturated solutions exhibited a distinct cathodic peak, confirming ORR activity. This comparison validates that the observed current is exclusively due to ORR, not capacitive effects or side reactions. Furthermore, double-layer capacitance (*C*_dl_) calculations (Figure 4d) revealed that 6 NaBH_4_–OMS­(2) exhibited the highest *C*_dl_ value (5.8 mF·cm^–2^) compared to pristine OMS(2) *C*_dl_ (3.9 mF cm^–2^). This enhanced *C*_dl_ directly correlates with a larger electrochemical surface area (ECSA) for 6 NaBH_4_–OMS­(2), as ECSA is proportional to *C*_dl_ (ECSA = *C*_dl_/*C*_s_, where *C*_s_ is the specific capacitance of the material) (Figure S3). This suggests that optimized oxygen vacancy concentration not only enhances intrinsic catalytic activity but also increases available reaction sites, thereby contributing to its superior performance, ultimately emphasizing the critical importance of meticulously controlling O_2_ vacancy defects to optimize the catalytic performance of OMS(2) for ORR applications.

**Figure 4:**
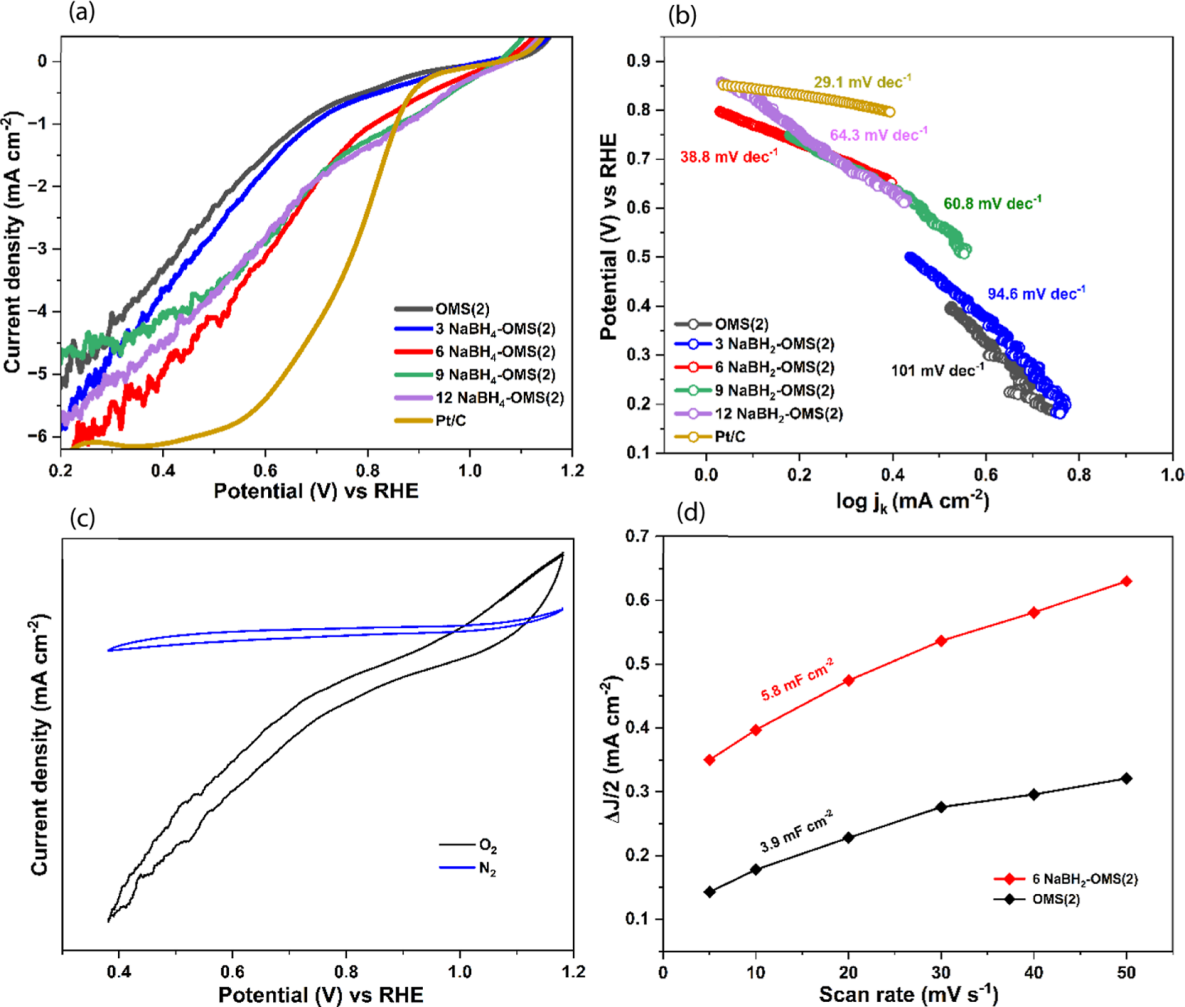
(a) LSV curves at 5 mV s^–1^ (b) Tafel plot of all electrocatalysts. (c) CV graph of ORR activity in O_2_ and N_2_ saturated 0.1 M KOH electrolyte at 10 mV s^–1^ (d) *C*_dl_ value of OMS(2) and 6 NaBH_4_–OMS­(2) electrocatalysts.

The electrochemical performance of the 6 NaBH_4_–OMS­(2) electrocatalyst was further evaluated through RDE, linear sweep voltammetry (LSV) at varying rotation speeds­(400–2400 rpm) at a scan rate of 5 mV s^–1^, as depicted in Figure 5a. The results showed a positive correlation between the rotating speed and the current density, indicating enhanced mass transport and catalytic efficiency.^35–37^ It was observed that a limited increase in the current density with increasing rotation speed from 400 to 2400 rpm, which indicates the ORR is not primarily limited by the external mass transport of oxygen from the bulk electrolyte to the electrode surface. Instead, the reaction rate appears to be primarily governed by intrinsic surface reaction kinetics at the catalyst/electrolyte interface, or by internal mass transport limitations within the catalyst layer, such as the diffusion of oxygen or protons to deeply embedded active sites. To elucidate the electron transfer mechanism, the Koutecky–Levich (K–L) plot was constructed, as shown in Figure 5b. The plots exhibit excellent linearity across the measured potential range, confirming the high applicability of the K–L model to our system. The calculated electron transfer number (*n*) was consistently near 4, strongly indicating a preference for the direct 4-electron ORR pathway. A minor, yet persistent, deviation from ideal linearity can be discerned at the highest rotation speeds (the leftmost data points). This is a well-documented and intrinsic characteristic of high-surface-area, porous electrocatalysts.^38^ The ideal K–L model assumes a perfectly flat 2D electrode, whereas our nanostructured material has a porous 3D architecture. At high rotation speeds, the reaction rate can become partially limited by the slower diffusion of O_2_ within the catalyst’s internal pores. This additional internal mass transport resistance is responsible for the slight deviation from the ideal model. Electrochemical impedance spectroscopy (EIS) was conducted to gain further mechanistic insights into the improved ORR performance, with the Nyquist plots presented in Figure 5c and fitted using the equivalent circuit model shown in the inset. Analysis of the EIS data revealed a significantly smaller semicircle diameter for 6 NaBH_4_–OMS­(2) compared to pristine OMS(2). Quantitative fitting using the equivalent circuit model (Figure 5c, inset) confirms this observation, yielding a charge transfer resistance (*R*_ct_) of just 30.08 Ω for the optimized catalyst, a value substantially lower than the 56.03 Ω obtained for pristine OMS(2). A lower *R*_ct_ signifies faster electron transfer kinetics at the catalyst–electrolyte interface, which is a crucial factor for enhanced electrocatalytic activity.^39^ This finding from EIS directly corroborates the superior ORR performance observed in the LSV curves (Figure 4a), where 6 NaBH_4_–OMS­(2) exhibited a higher current density and a more positive half-wave potential. The reduced charge transfer resistance for 6 NaBH_4_–OMS­(2), therefore, highlights that the optimized oxygen vacancy concentration effectively facilitates the charge transfer steps during the ORR, leading to improved overall reaction kinetics. Moreover, the catalyst demonstrated exceptional stability. As evidenced by chronoamperometry at 1600 rpm (Figure 5d), the optimized catalyst maintained 90% of its initial current density after a prolonged 10 h test. In stark contrast, the pristine OMS(2) retained only 71% of its activity under the same conditions. This result underscores the significantly enhanced durability of the treated material, highlighting its potential for durable electrochemical applications.

**Figure 5:**
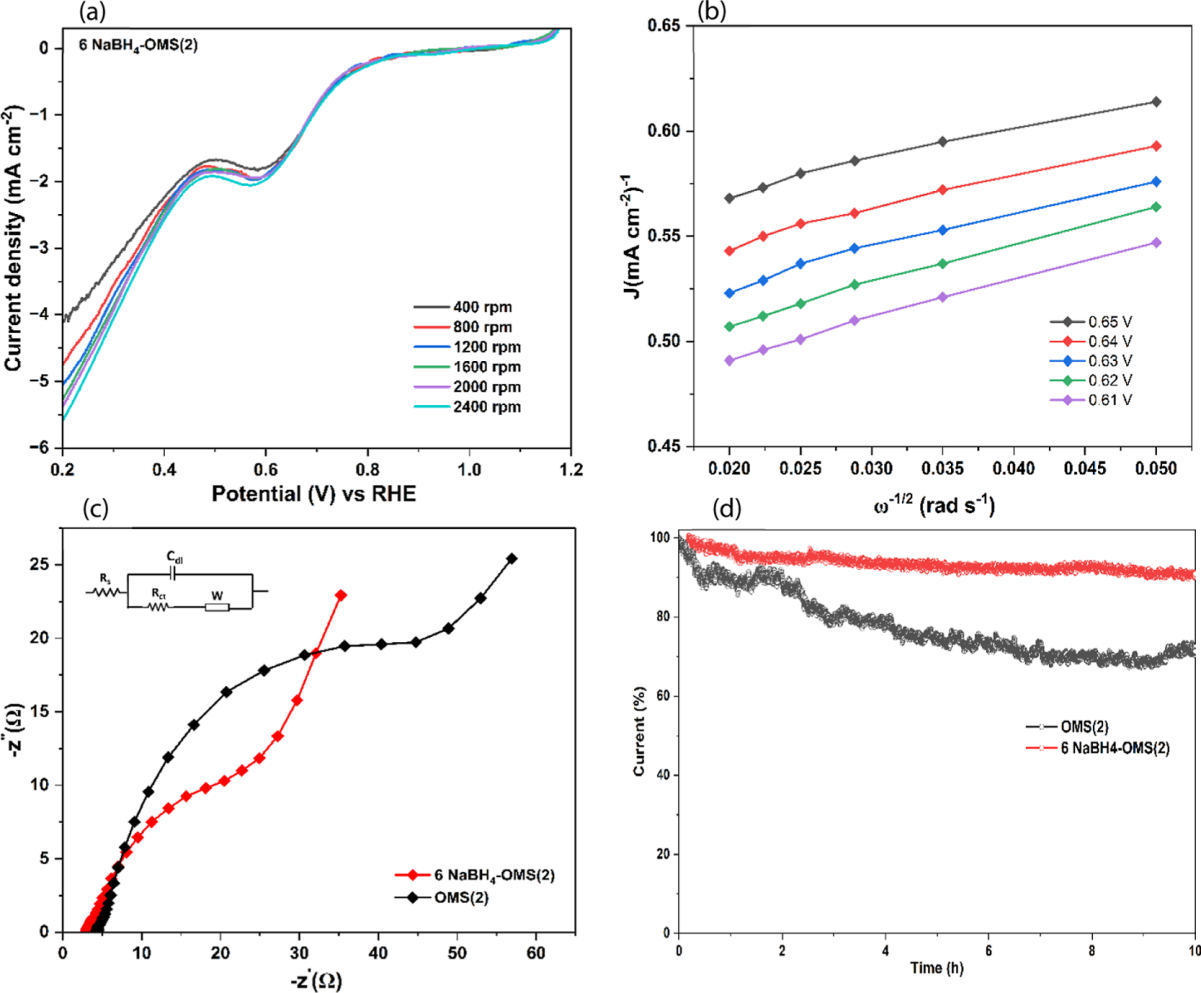
(a) LSV curves at a scan rate of 5 mV s^–1^ (b) corresponding to K-L plots of 6 NaBH_4_–OMS­(2) electrocatalyst. (c) EIS graph (inset equivalent model) (d) chronoamperometric stability of OMS(2) and 6 NaBH_4_–OMS­(2) electrocatalysts.

To elucidate the catalyst’s remarkable durability, a comprehensive post-mortem characterization was conducted after a 10 h chronoamperometric test. FESEM analysis (Figure S4) confirmed that the catalyst largely retained its original nanosphere morphology, maintaining a consistent average particle size of approximately 76.9 nm (Figure S5). This indicates excellent structural integrity with minimal aggregation or dissolution at the macroscopic level. Complementary EDS analysis revealed only a slight increase in the oxygen atomic percentage, further attesting to the material’s high compositional stability under demanding operational conditions. Concurrently, XPS analysis revealed dynamic surface evolution. The O 1s spectra showed a decrease in both oxygen vacancies (O_2_) and lattice oxygen (O_1_), alongside a significant increase in chemisorbed oxygen (O_3_) (Figure S6). This suggests a surface reconstruction where the original lattice is partially consumed but re-equilibrates into a more hydroxylated or oxygenated surface phase. Furthermore, a slight reoxidation was observed in the Mn 2p spectra, with the Mn^3+^ content subtly decreasing from 70.1% to 67% and Mn^4+^ increasing from 29.9% to 33% (Figures S7 and S8).^40,41^ This combined evidence from FESEM and XPS demonstrates that the catalyst’s high current retention is supported by its ability to maintain structural integrity while undergoing a beneficial dynamic surface reconstruction, forming a stable active interface under continuous ORR operation.

## 4 Conclusion

This study demonstrates the successful modification of octahedral molecular sieve (OMS-2) electrocatalysts through sodium borohydride (NaBH_4_) treatment, which significantly enhances their oxygen reduction reaction (ORR) performance. The NaBH_4_ treatment introduces oxygen vacancies and alters the Mn^3+^/Mn^4+^ redox couple, crucial for improving catalytic activity. The optimal concentration of NaBH_4_ (6 mmol/L) yields the highest current density and most favorable reaction kinetics, as evidenced by linear sweep voltammetry (LSV) and Tafel plot analysis. The enhanced electrochemical surface area (ECSA) and electron transfer number (approaching 4) further contribute to the superior performance of the 6 NaBH_4_–OMS­(2) catalyst. Notably, this catalyst exhibits excellent stability, retaining 90% of its current density after a 10 h durability test. These findings highlight the importance of controlled oxygen vacancy creation in optimizing the ORR activity of OMS(2) electrocatalysts.

## Supplementary Material



## Data Availability

Data used is available throughout the manuscript text and Supporting Information file.

## Notes

Physical and electrochemical characterization details; particle size distribution histograms of OMS(2) and 6 NaBH_4_–OMS­(2) before and after stability testing; FESEM image, EDS and XPS spectra of 6 NaBH_4_–OMS­(2) after stability; EDS elemental composition tables of OMS(2) and 6 NaBH_4_–OMS­(2) before and after stability; and a electrochemical comparative analysis of 6 NaBH_4_–OMS­(2) and OMS(2) (PDF)
