# Supplementary material for: Surface-Engineered Manganese Oxide via Sodium Borohydride for Optimized ORR Active Electrocatalyst
Source: ACS Omega. 2025 Oct 23;10(43):51882–90. doi: 10.1021/acsomega.5c08148 (PMC12594001; doi:10.1021/acsomega.5c08148)
Supplement: Supplementary file 1 [file ao5c08148_si_001.pdf]

Surface-Engineered Manganese Oxide via Sodium Borohydride for Optimized ORR Active Electrocatalyst

Jithul KP[a], Jay Pandey[a]*

[a]*Department of Chemical Engineering, Birla Institute of Technology & Science Pilani, Pilani Jhunjhunu-333031, Rajasthan, India*

Corresponding author: Tel: +91-1596 255883, E-mail: jay.pandey@pilani.bits-pilani.ac.in

Physical and electrochemical characterization

The morphology and microstructure of the developed catalyst were determined with field emission scanning electron microscopy (FE-SEM), FEI Apreo LoVac and transmission electron microscopy (TEM, Thermo Scientific Talos F200C TEM). Energy-dispersive X-ray spectroscopy (EDS) was the instrument used to determine the composition. Structural identification via X-ray diffraction (XRD) using a Rigaku ULTIMA-IV. The identification of molecular bonds was carried out using Fourier transform infrared spectroscopy (FT-IR) on a Perkin Elmer instrument. Using a Thermo Fisher Scientific UK K-Alpha spectrometer, X-ray photoelectron spectroscopy (XPS) was used to determine the composition of elements as well as chemical bonding. For XPS analysis, Gaussian peak fitting was used for curve deconvolution. Raman spectroscopy, conducted on a LAB RAM HR Horiba system, provided further insight into the molecular bonding and assessed the degree of graphitisation within the materials.

Electrocatalytic performance was assessed using a 3-electrode electrochemical cell connected to a BioLogic SP-50e potentiostat. Ag/AgCl electrode served as the reference, a Pt wire as the counter electrode, and the fabricated electrocatalyst as the working electrode. To standardise the data, all potentials were converted to the RHE scale using the equation: $E_{(RHE)} = E_{(Ag/AgCl)} + 0.197 + 0.0591 \times pH$. Linear sweep voltammetry (LSV) was conducted in a 0.1 M KOH electrolyte (pH 13) at a scan rate of 5 mV/s. Electrochemical impedance spectroscopy (EIS) was performed over a frequency range from 0.1 Hz to 100 kHz. The electrochemical active surface area (ECSA) was determined by cyclic voltammetry (CV) through analysis of the double-layer capacitance (Cdl) at various scan rates.

The Koutecky-Levich equation, used to analyse rotating disk electrode (RDE) data, describes the correlation between current and rotational velocity. The equation can be expressed as:

$$1/i = 1/i_k + 1/i_d \quad (1)$$

where I represents the measured current, i_k is the kinetic current, and i_d is the diffusion-limiting current.

The diffusion-limiting current (i_d) is given by:

$$i_d = 0.62nFC_0D_0^{2/3}\nu^{-1/6}\omega^{1/2} \quad (2)$$

where:

'n' represents electrons transferred per O_2.
'F' is Faraday's constant.
'C_0' is oxygen's bulk concentration.
'D_0' is oxygen's diffusion coefficient.
'ν' is electrolyte kinematic viscosity.
'ω' is electrode angular rotation (rad/s).

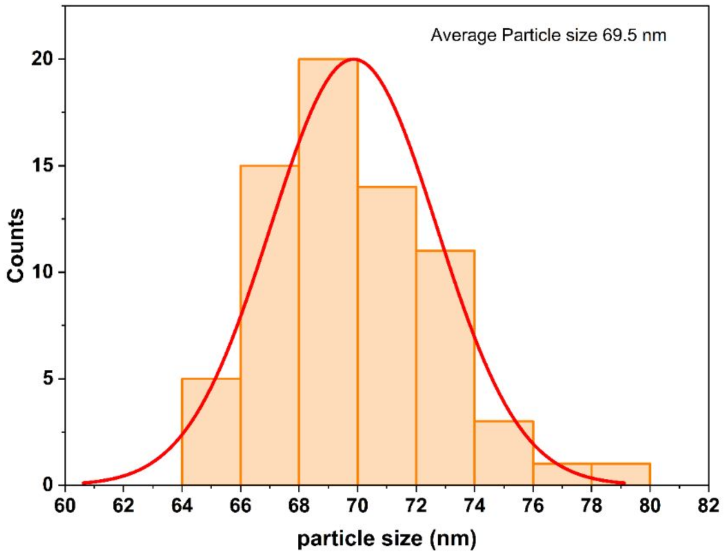

Figure S1. OMS(2) electrocatalyst particle size distribution histogram.

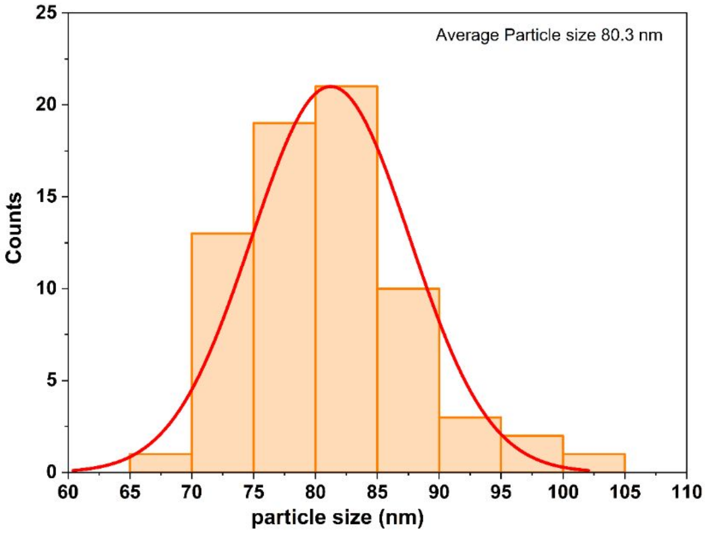

Figure S2. 6 $NaBH_4$- OMS(2) electrocatalyst particle size distribution histogram.

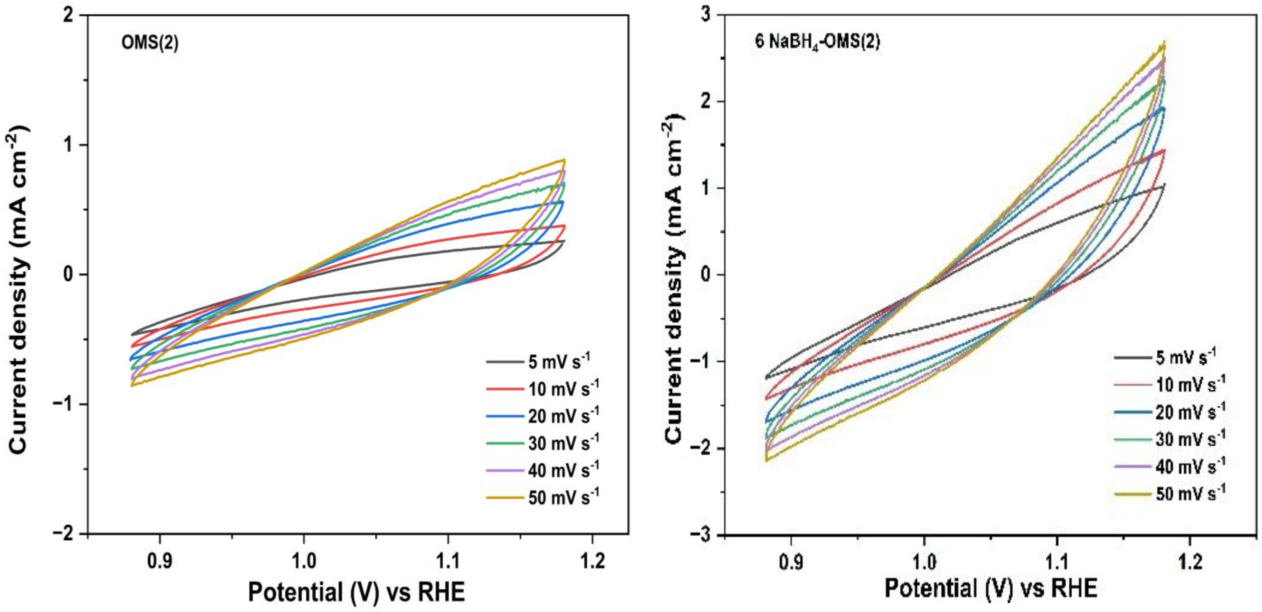

Figure S3. CV graphs of OMS(2) and 6 $NaBH_4$- OMS(2) electrocatalysts for C_{dl} value calculation.

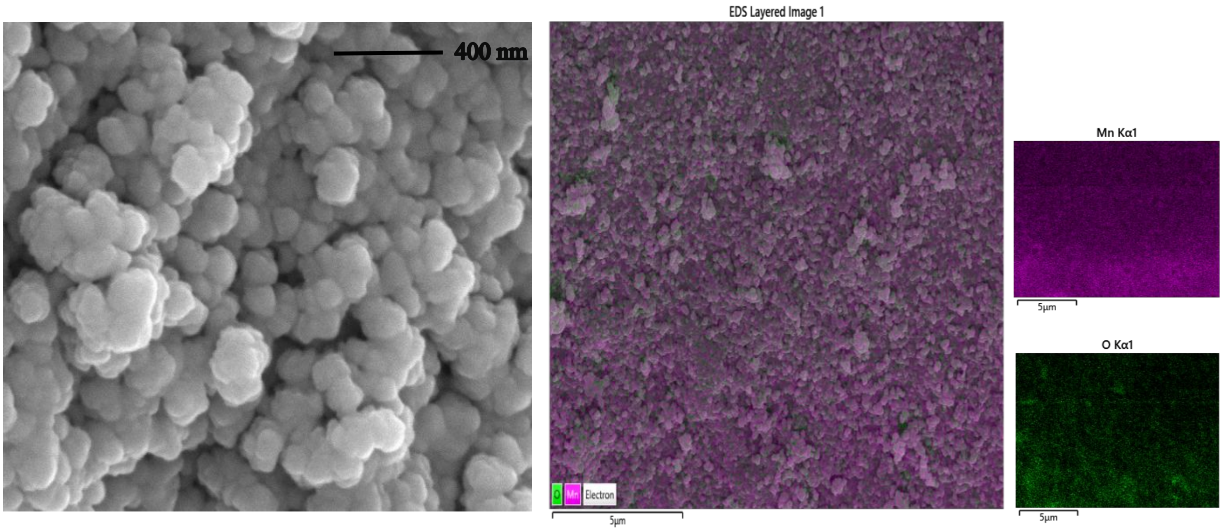

Figure S4. FESEM image and EDS mapping of 6 $NaBH_4$- OMS(2) electrocatalyst after stability test.

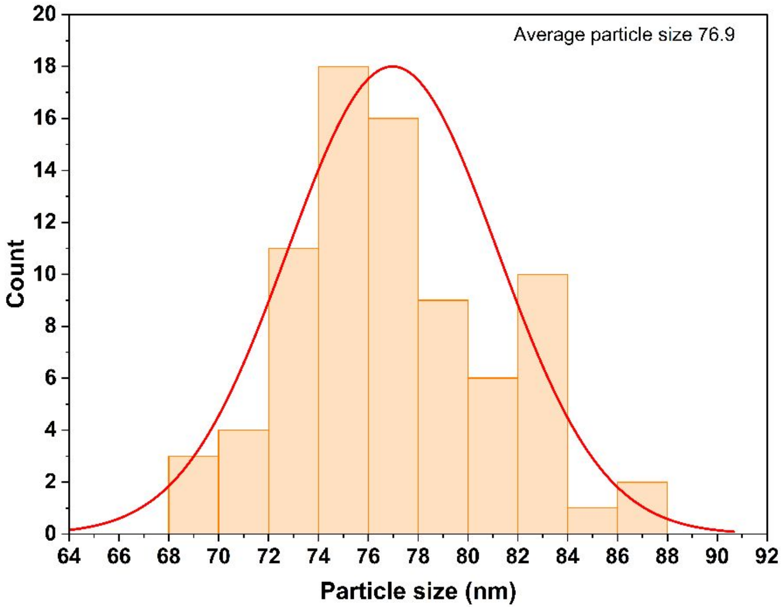

Figure S5. 6 $NaBH_4$- OMS(2) electrocatalyst particle size distribution histogram after stability test.

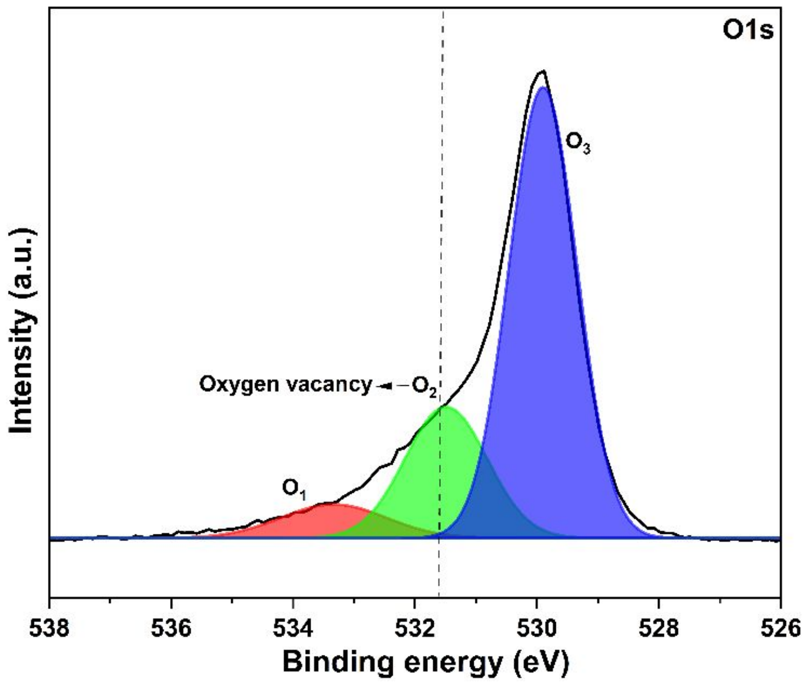

Figure S6. XPS image of O1s spectrum of 6 $NaBH_4$-OMS(2) electrocatalyst after stability test.

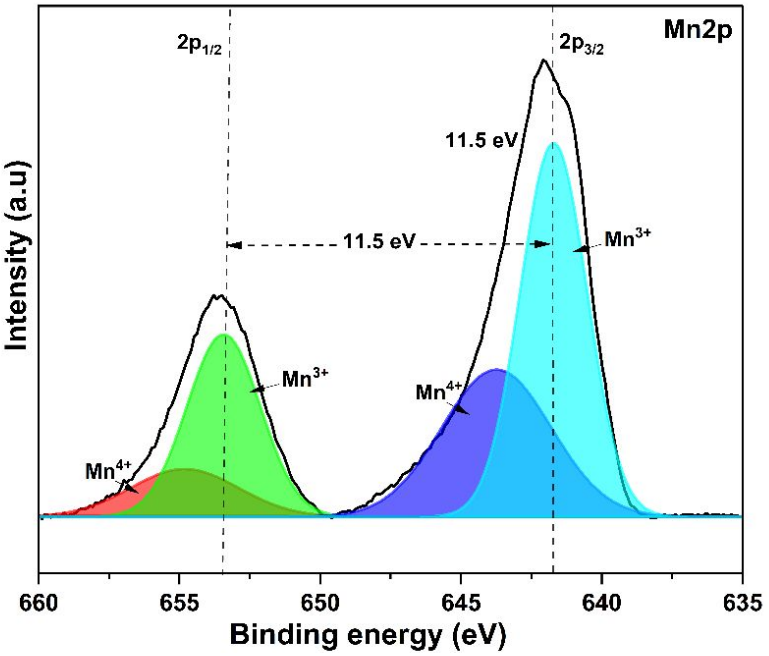

Figure S7. XPS image of Mn2p spectrum of 6 $NaBH_4$-OMS(2) electrocatalyst after stability test.

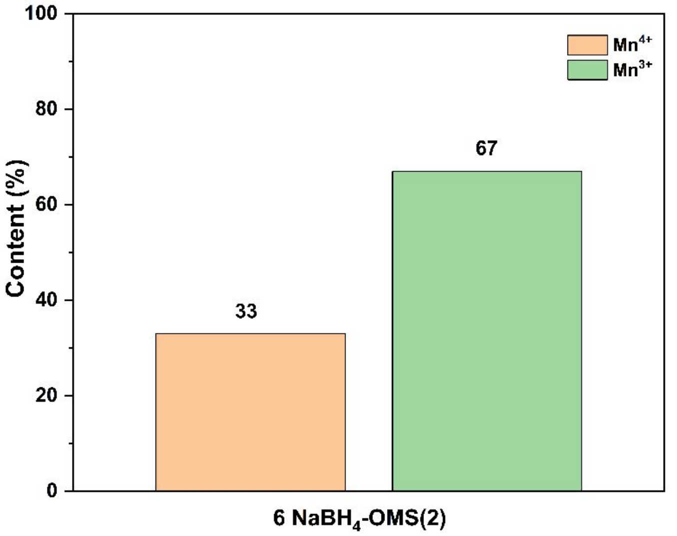

Figure S8. Relative content of Mn^{3+} and Mn^{4+} of 6 $NaBH_4$-OMS(2) electrocatalyst after stability test

Table S1. EDS elemental composition of OMS(2), 6 $NaBH_4$-OMS(2) before and after stability electrocatalysts.

Electrocatalyst	Elements	Weight %	Atomic %
OMS(2)	Mn	92.26	77.63
	O	7.74	22.37
6-$NaBH_4$-OMS(2)-before stability test	Mn	95.33	85.60
	O	4.67	14.40
6-$NaBH_4$-OMS(2)-after stability test	Mn	93.76	81.40
	O	6.24	18.60

Table S2. Comparative analysis of 6 $NaBH_4$-OMS and OMS(2) electrocatalysts with a state-of-the-art electrocatalyst.

Catalyst	$E_{ORR\text{-}Onset}$/V	$E_{ORR\text{-}1/2}$/V	j_{max}/mA cm^{-2}
**OMS(2)**	1.02	0.5	5
**6 $NaBH_4$-OMS(2)**	1.06	0.66	6
**Pt/C**	1.1	0.76	6.1
